# Supplementary material for: Quantitative Determination of Aflatoxin B1 in Maize and Feed by ELISA and Time-Resolved Fluorescent Immunoassay Based on Monoclonal Antibodies
Source: Foods. 2024 Jan 19;13(2):319. doi: 10.3390/foods13020319 (PMC10815167; doi:10.3390/foods13020319)
Supplement: Supplementary file 1 [file foods-13-00319-s001.zip › foods-2803003-supplementary.pdf]

Quantitative determination of AFB_1 in corn and feed by ELISA and time-resolved fluorescent immunoassay based on monoclonal antibodies

Shiyun Han[1#], Yalin Yang[1#], Ting Chen[1#], Guyue Cheng[2#], Bijia Yang[1], Yanfei Tao[2], Xiaohui Fan[3*], Haihong Hao[2*], Dapeng Peng[1*]

1. Synthesis of hapten AFB1-CMO

Methodology for the synthesis of AFB1 hapten.

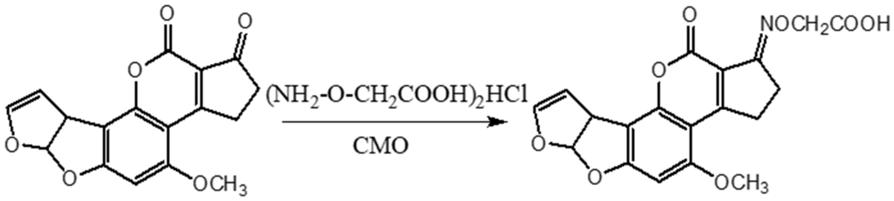

Figure S1. Synthesis of AFB_1-CMO

2. Characterization of haptens

As shown in Figure S2, AFB1-CMO was successfully synthesized.

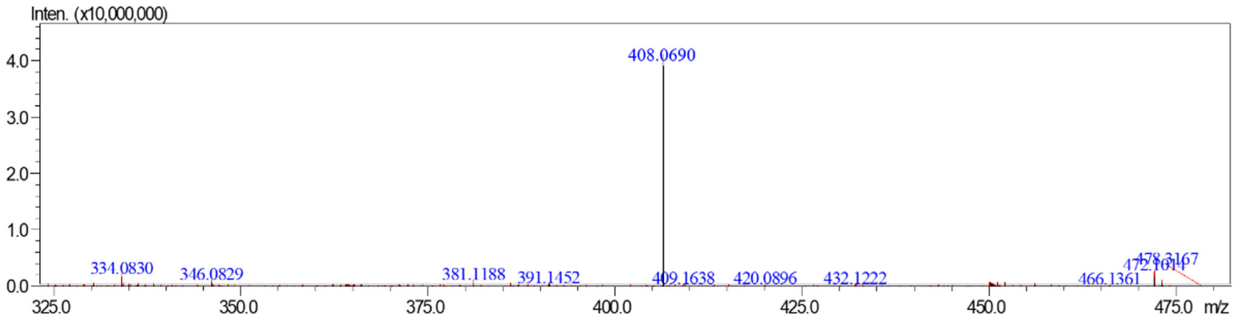

Figure S2. MS spectra of hapten AFB_1-CMO, calculated for $C_{17}H_{12}O_6$ [M-Na]+ 408.0690, found 408.0690

3.Identification of antiserum and monoclonal antibody

Mice with immunogen AFB_1-DCC-KLH and immune dose of 80 μg were selected for cell fusion. The results are shown in Table S2, and cell 3B9 was selected for subcloning.

Table S1 The effect of immunizing dose

Immunogen	Time	Dose (μg)	Titre (1:X)				IC_{50} (μg/L)
			Mouse1	Mouse2	Mouse3	Mouse4	AFB_1
		50	-	200	200	-	5
AFB_1-DCC-KLH	3	80	128000	6400	128000	128000	2.5
		100	death	death	death	death	-
		50	32000	32000	64000	32000	-
AFB_1-EDC-HSA	3	80	256000	128000	256000	256000	-
		100	death	death	death	death	-

Note: "-" indicates invalid price/specificity.

Table S2 The screening of hybridoma cell

Cells	OD of Blank	OD of AFB1 (0.25 μg/L)	Inhibition ratio (%)
3B9	2.75	1.41	51%
1B6	1.93	0.813	42%

The subclasses and light chains of the 3B9 were characterized using a rapid ELISA isotype kit with mouse (monoclonal antibody Mab). The detection results are shown in TableS3. According to the determination index of the kit, OD value > 0.2 is considered positive, then 3B9 class is IgG1 and light chain is Kappa chain.

Table S3 Identify of sub-type of monoclonal antibody produced by AFB1/3B9

Class	IgG1	IgG2a	IgG2b	IgG3	IgA	IgM	Kappa	Lambda
OD value	0.52	0.11	0.15	0.053	0.091	0.064	0.54	0.067

Heterogeneous format could result in antibody with higher affinity towards the analyte than the coating antigen or tracer hapten in some cases. Therefore, in order to achieve sensitivity results, heterology coating is preferred than homologous format. In this study, as show in Table S6, linkage of AFB1-EDC-OVA was used as heterogeneous coating antigens. The optimal conditions for ic-ELISA were determined as a concentration of 1.08 μg/mL of coating antigen (AFB1-EDC-OVA) with a 1:1500 antibody (3B9) dilution.

Table S4 Optimization of coating antigen concentration

coating antigen concentration(μg/mL)	antibody dilution ratio(1:X)	OD of Blank	IC_{50} (μg/L)
1.08	1500	1.874	0.0498
0.542	750.0	2.087	0.0720

Table S5 Optimization of dilute strength

antibody dilution ratio(1:X)	OD of Blank	IC_{50} (μg/L)
1200	2.01	70.2
1500	2.09	39.1
1800	2.01	53.1
2100	1.98	65.4
2400	2.02	76.8

Table S6 The effect of coating antigen to antibody sensitivity

coating antigen	antibody dilution ratio(1:X)	OD of Blank	IC_{50} (μg/L)
AFB1-EDC-OVA (1.08 μg/mL)	1500	2.055	0.04503
AFB1-EDC-BSA (0.54μg/mL)	750	2.032	0.07308

As shown in Table S7, CR of AFB1, AFB2, AFG1 and AFG2 were 100%, 7.3%, 3.1% and 2.7%, respectively. The results showed that the antibody had high specificity.

Table S7 The cross reactivities with other AFTs.

AFTs	IC_{50} (ng/L)	Cross-reactivity (%)
AFB_1	0.0386	100
AFB_2	0.529	7.31
AFG_1	1.27	3.04
AFG_2	1.46	2.64

4. Preparation of TRFM labeled antibodies

TRFM were linked to mAb by activated ester method. In short, 0.1 mg of TRFM was added to 0.5 mL MES buffer (50 mmol/L, pH 6.0), centrifuged at 12,000 rpm for 20 min, and then ultrasonic in MES buffer for 10 s. The active carboxyl groups on the surface of TRFMs were mixed by adding 40 μL NHS (1mg/mL) and 40 μL EDC (1mg/mL) in turn. After activation for 20 min, centrifugation at 12000 rpm for 20 min at 4 °C. The supernatant was discarded, and the precipitate was dissolved in 0.5 mL

boric acid buffer (pH 8.0), and then dispersed by ultrasound for 10s. Then 10 μL of AFB1-mAb (1 mg/mL) was added and incubated for 3 h, then 50 μL of 10% BSA was added to block the reaction for 3 h. Finally, the solution was centrifuged at 12,000 rpm for 10 min at 4 ℃. The supernatant was discarded, and the residue was reconstituted by 100 μL eddy current mixing and ultrasonic re-suspension. Store the suspension at 4 ℃ for later use.

5.Optimization of TRFICA

The optimization results of the binding pad are shown in Figure S3a and b. The fluorescent probe on the polyester fiber bonding pad has better and more consistent release effect, and the detection background is cleaner, so the bonding pad is made of polyester fiber.

The optimization results of NC membranes are shown in Figure S3c. There was no residue of fluorescent probe in the overlap of PALL 170 type NC membranes and the binding pad, the detection background was clean, the C-line and T-line were complete, and the difference between negative and positive was obvious. Therefore, PALL 170 type NC membranes is selected.

The optimization results of detection time are shown in Figure S3d. At 2-6 min, the fluorescence intensity and inhibition rate of the fluorescent test paper increased gradually, indicating that the chromatographic reaction was in progress. At 6 min, the fluorescence intensity and inhibition rate reached the maximum value, and remained almost unchanged after that. Therefore, incubation at 37 ℃ for 6 min was used as the best detection time for the two kinds of fluorescent test paper.

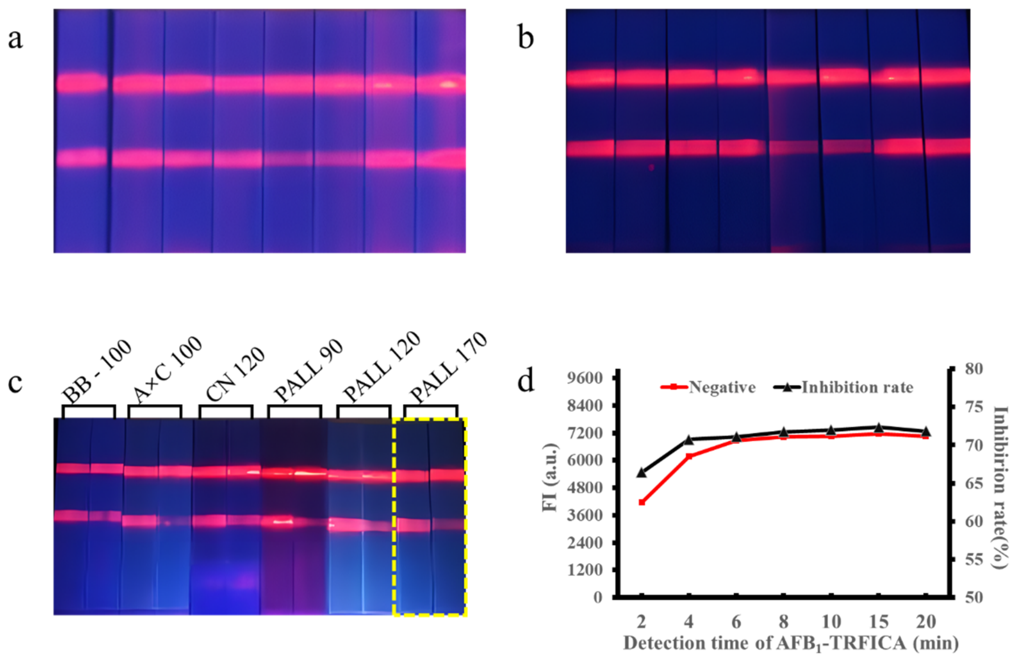

Figure S3. TRFICA optimization results. (a) Binding pad material screening results: glass fiber. (b) Binding pad material screening results: polyester fiber. (c)Screening results of NC membrane. (d) Optimal results for detection time of AFB_1-TRFICA.

The optimization results of the sample pad are shown in Table S8. When the sample pad model is MA0120, the fluorescence intensity is the highest and the coefficient of variation is the smallest, indicating that the detection result is more accurate, so the model MA0120 is selected as the sample pad.

Table S8 Results of sample pad screening.

Sample pad model	Mean fluorescence intensity	T/C value Mean value	Coefficient of variation(CV%)
GF2-S	7284.4	0.94	17.5
Fusion 6	9251.2	1.1	26.1
MA0120	13023	1.4	4.17

The optimization results of concentration optimization of AFB_1-BSA and goat anti-mouse IgG were shown in Table S8. Similar fluorescence intensity was measured when the antigen concentration was 0.5 mg/mL and 0.3 mg/mL. However, too high T-line antigen concentration or too low C-line concentration will cause the T/C value to be too large and the detection gradient is not obvious. Therefore, the coated concentration of antigen and goat anti-mouse IgG was selected to be 0.3 mg/mL.

Table S8 Optimization coating concentration of AFB_1-BSA and goat anti-mouse IgG.

Antigen on T line (mg/mL)	C line (mg/mL)	AFB_1 (μg/L)	T-line fluorescence intensity	T/C value Mean value	Inhibition rate (%)
0.5	0.3	0	13683	2.242	73.36
		2	6861.5	0.5974	
	0.2	0	15035	2.834	66.95
		2	9041.2	0.9368	
0.3	0.3	0	15912	1.913	70.14
		2	8667.3	0.5704	
	0.2	0	13901	2.674	70.79
		2	7400.7	0.7812	

6.Valuation and comparison of ic-ELISA and time-resolved fluorescent immunochromatographic assay (TRFICA)

The blank corn samples were detected by HPLC-MS/MS, ic-ELISA and TRFICA simultaneously.

Table S9 Ic-ELISA and LC-MS/MS were used to detect AFB1 residues in corn.

Concentration (ng/L)	ic-ELISA (ng/L)		LC-MS/MS (ng/L)	
	1	2	1	2
50	43.58	48.69	38.35	37.22
100	98.66	94.52	80.52	81.37
150	143.5	151.3	139.3	137.8
200	185.7	178.9	173.1	172.3
250	243.6	241.3	236.2	240.5
300	306.3	281.3	280.6	285.9

Table S10 Ic-ELISA and TRFICA were used to detect AFB1 residues in corn.

Concentration (μg/L)	Ic-ELISA (μg/L)		TRFICA(μg/L)	
	1	2	1	2
0.1	0.1173	0.1319	0.09131	0.1306
0.25	0.1732	0.1931	0.2375	0.2485
0.5	0.4112	0.3676	0.5229	0.4491
1	0.7577	0.7099	1.035	0.8905
2	1.944	1.647	2.115	2.305
3	2.664	2.776	3.133	2.647
